# Electro-active metaobjective from metalenses-on-demand

**DOI:** 10.1038/s41467-022-34494-0

**Published:** 2022-11-23

**Authors:** Julian Karst, Yohan Lee, Moritz Floess, Monika Ubl, Sabine Ludwigs, Mario Hentschel, Harald Giessen

**Affiliations:** 1 4th Physics Institute and Research Center SCoPE, University of Stuttgart, Pfaffenwaldring 57, 70569 Stuttgart, Germany; 2 IPOC-Functional Polymers, Institute of Polymer Chemistry, & Center for Integrated Quantum Science & Technology (IQST), University of Stuttgart, Pfaffenwaldring 55, 70569 Stuttgart, Germany

**Keywords:** Nanophotonics and plasmonics, Optical materials and structures, Electronics, photonics and device physics, Nanoscale materials

## Abstract

Switchable metasurfaces can actively control the functionality of integrated metadevices with high efficiency and on ultra-small length scales. Such metadevices include active lenses, dynamic diffractive optical elements, or switchable holograms. Especially, for applications in emerging technologies such as AR (augmented reality) and VR (virtual reality) devices, sophisticated metaoptics with unique functionalities are crucially important. In particular, metaoptics which can be switched electrically on or off will allow to change the routing, focusing, or functionality in general of miniaturized optical components on demand. Here, we demonstrate metalenses-on-demand made from metallic polymer plasmonic nanoantennas which are electrically switchable at CMOS (complementary metal-oxide-semiconductor) compatible voltages of ±1 V. The nanoantennas exhibit plasmonic resonances which can be reversibly switched ON and OFF via the applied voltage, utilizing the optical metal-to-insulator transition of the metallic polymer. Ultimately, we realize an electro-active non-volatile multi-functional metaobjective composed of two metalenses, whose unique optical states can be set on demand. Overall, our work opens up the possibility for a new level of electro-optical elements for ultra-compact photonic integration.

## Abstract (web summary)

Future optical devices, e.g., for AR and VR, will require sophisticated flat metaoptics with unique optical functionalities. The authors demonstrate a metaobjective based on electrically switchable metallic polymer metalenses, whose optical states and focal length is adjustable via CMOS compatible voltages.

## Introduction

The miniaturization of optical components and their integration into functional small-scale electro-optical devices is tremendously fueled by advances in the field of active nanoantennas and metasurfaces^1,2^. The reason lies with their unique feature to manipulate light-matter interaction on sub-wavelength scales^3–8^. The functionalities of (active) metasurfaces range from beam steering devices for LiDAR (Light Detection and Ranging)^9–13^, to (dynamic) metalenses^14–21^, metasurfaces for VR (virtual reality) as well as AR (augmented reality) applications^22–28^, or nanophotonic spatial light modulators^9,29,30^. Especially, switchable and active metalenses are of interest for the miniaturization and for extending the functionality of complex and integrated on-chip imaging systems. Typically, bulky lenses or mechanically adjustable zoom-objectives are necessary, while active metalenses allow similar adjustments to, e.g., the focal length on ultra-small length scales without any required mechanical adjustment. In detail, the active switching of metalenses relies on the change of the optical properties of the used materials upon an external stimulus. Most prominent, these transitions are either driven by temperature^15,16,31^, chemically^32–36^, or electrically^11,17,37–42^, with the latter being the most desirable for electro-optical devices.

However, most current approaches of active metalenses do not electrically switch the nanoantenna material itself. In fact, either the material surrounding the metalens or the metalens material itself is dynamically tuned/altered thus limiting the achievable switching contrast, functionality, and overall design freedom^17,19,20,43^. In contrast, using nanoantennas from smart functional materials with electrically driven metal-to-insulator transitions would allow to turn a metalens and thus its functionality fully ON or OFF on demand. Here, we demonstrate, for the first time, active metalenses-on-demand and a multi-functional metaobjective composed of electrically switchable metallic polymer nanoantennas. The plasmonic metalenses are switchable between fully ON- and OFF-states via an applied voltage of only ±1 V. A detailed comparison of our approach for such electro-active metasurfaces/-lenses to existing work discussed above is shown in Table S1 in the Supplementary Information.

## Results and discussion

**Fig. 1:**
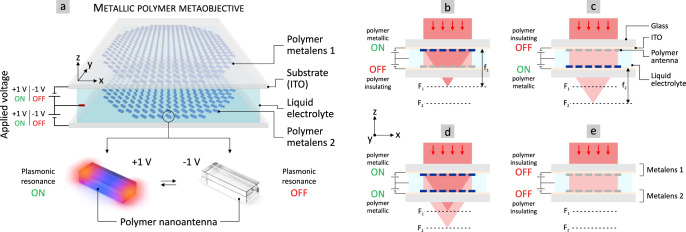
Concept of electrically switchable metalenses-on-demand and multi-functional metaobjective from metallic polymer. **a** Top: Schematic illustration of a metaobjective comprising two electrically switchable metalenses on ITO (indium-tin-oxide) covered substrates. The metalenses consist of electrically switchable metallic polymer nanoantennas. An electrolyte is used as separation and to allow electrochemical switching. The refractive power of the polymer metalenses is switched ON or OFF on demand via an applied voltage of only +1 V or −1 V, respectively. Bottom: The electrical switching is based on a reversible metal-to-insulator transition of the metallic polymer. A voltage of +1 V turns polymer nanoantennas metallic and their plasmonic resonance ON. A voltage of −1 V switches the polymer nanoantennas into an insulating state and their plasmonic resonance OFF. **b**–**e** Depending on the individual voltage applied to the polymer metalenses, four different states become possible. **b** (metalens 1: ON, metalens 2: OFF): focus at ${{{{{{\rm{F}}}}}}}_{1}$. **c** (OFF, ON): focus at ${{{{{{\rm{F}}}}}}}_{2}$. **d** (ON, ON): focus at ${{{{{{\rm{F}}}}}}}_{1}$ and ${{{{{{\rm{F}}}}}}}_{2}$. **e** (OFF, OFF): no focus.

The concept of our metallic polymer metalenses and metaobjective is illustrated in Fig. 1. The objective consists of two independent electrically switchable metalenses (polymer metalens 1 and 2), placed on an ITO (indium-tin-oxide) coated substrate and separated by an electrolyte (Fig. 1a). Each metalens comprises plasmonic polymer nanoantennas which are switchable between a metallic and insulating state via applied voltages of +1 V and −1 V, respectively. This is illustrated in the lower part of Fig. 1a. In particular, the plasmonic resonance of the nanoantennas can be switched fully ON or OFF electrically. The underlying mechanism is an electrochemically driven optical metal-to-insulator transition in the metallic polymer PEDOT:PSS (poly(3,4-ethylenedioxythiophene):poly(-styrene sulfonate)) (see Fig. S1 in the Supplementary Information for the dielectric functions of PEDOT:PSS in the metallic and insulating state)^11^. This concept is rather different from other direct approaches using nanoantennas from phase-change materials such as, e.g., GST (Germanium-Antimony-Telluride)^37,38^. These materials are typically switched by temperature, while the transitions employ a structural reorientation (e.g., amorphous to crystalline state) and thus a shift of the refractive index instead of a metal-to-insulator transition. These temperature-assisted switching mechanisms can be speed-up significantly by using resistive heating, however, this requires comparably high voltages (5–25 V)^37,38^. Consequently, in our concept a CMOS (complementary metal-oxide-semiconductor) compatible voltage of +1 V turns the nanoantenna building blocks into the metallic state and thus the individual metalens into the ON state—the refractive power of the lens is switched ON. A voltage of −1 V turns the metalens and thus the refractive power OFF. As mentioned above, a detailed comparison of our concept to other approaches is summarized in Table S1 in the Supplementary Information.

In the combined case of the metaobjective, we obtain four different output states, depending on the voltage applied to the individual polymer metalenses. These multiple states are depicted in Fig. 1b–e. In Fig. 1b, metalens 1 is turned ON (+1 V) while metalens 2 is turned OFF (−1 V). Only metalens 1 focuses the incoming collimated light beam. The metaobjective possesses a focal length ${{{{{{\rm{f}}}}}}}_{1}$. The inverted case in Fig. 1c (metalens 1: OFF, metalens 2: ON) causes only metalens 2 to focus the incoming light. The metaobjective possesses a focal length ${{{{{{\rm{f}}}}}}}_{2}$. In the third case (metalens 1: ON, metalens 2: ON), we obtain a unique multi-focal state. Due to the general working principle of our metalenses using a geometrical phase and circularly polarized light, the two metalenses do not influence each other. Incoming right-circularly polarized (RCP) light is focused as left-circularly polarized (LCP) light and vice-versa. This means that the focused light is cross-polarized with respect to the incident light. Both metalenses focus the incoming circularly polarized light separately and we obtain two foci, as depicted in Fig. 1d. In addition, the metallic polymer metaobjective can also be switched fully OFF. This last case with no focused light in Fig. 1e results from both metalenses to be switched OFF with an applied voltage of −1 V.

**Fig. 2:**
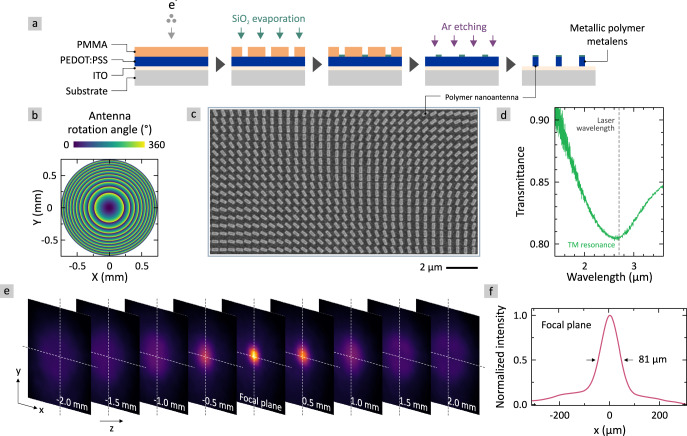
Fabrication and static characterization of a metallic polymer metalens. **a** Fabrication route to obtain the metallic polymer metalens comprising plasmonic nanoantennas. Details are given in the main text. **b** 2D map of nanoantenna rotation angle to obtain a quadratic phase profile of a metalens with focal length ${{{{{\rm{f}}}}}}$ = 5 mm, diameter of 1.5 mm, numerical aperture of 0.15, and illumination wavelength ${{{{{\rm{\lambda }}}}}}$ = 2.65 µm. **c** SEM image of a sub-area of the metallic polymer metalens showing the rotated nanoantennas. **d** TM plasmonic resonance of used metallic polymer nanoantennas (length 380 nm, width 160 nm, height 90 nm). Working laser wavelength (${{{{{\rm{\lambda }}}}}}$ = 2.65 µm) marked with dashed line. **e** Camera images at different ${{{{{\rm{z}}}}}}$-positions (along the optical axis) around the focal plane of the metallic polymer metalens. **f** Cross-section of beam profile in the focal plane of the metalens along ${{{{{\rm{x}}}}}}$-direction. The FWHM is 81 µm.

Before investigating the metaobjective, we characterize the static as well as dynamic optical properties of the individual metalens. The fabrication route is sketched in Fig. 2a: As substrate we use ITO (20 nm) covered glass to operate the final metadevices in transmission and allow electrical addressability. 90 nm of commercially available metallic polymer (PEDOT:PSS, Heraeus PH1000, Ossila) is spin-coated on the substrate and dried (120 °C, 15 min). The thin-film is covered with a two-layered poly(methyl-methacrylate) (PMMA) positive tone resist (Allresist AR-P 642.06 200k, Allresist AR-P 672.02 950k), used to define nanoantennas with electron beam lithography (EBL). After development in methylisobutylketone (MIBK), we use electron-gun evaporation to deposit a 30 nm silicon-dioxide (SiO_2_) layer. After lift-off, SiO_2_ serves as hard-mask for the subsequent argon (Ar) etching process, which removes uncovered PEDOT:PSS. As a result, we obtain a metallic polymer metalens from PEDOT:PSS.

The functionality and focal length of our metalenses depends on the curvature of the 2D quadratic phase profile, which relies on a geometric phase obtained via the geometrical arrangement and individual rotation of the nanoantennas. The rotation angle ${{{{{\rm{\varphi }}}}}}$ of a nanoantenna at position ${{{{{\rm{x}}}}}}$ and ${{{{{\rm{y}}}}}}$ is defined as

1 $$\varphi \left(x,y\right)=\frac{\pi }{{\lambda }_{0}}\left(\sqrt{{f}^{2}+{x}^{2}+{y}^{2}}-\left|f\right|\right)$$

where ${{{{{{\rm{\lambda }}}}}}}_{0}$ denotes the free space wavelength and ${{{{{\rm{f}}}}}}$ the focal length. The rotation angle in dependence of the position ${{{{{\rm{x}}}}}}$ and ${{{{{\rm{y}}}}}}$ for a metalens with ${{{{{\rm{f}}}}}}$ = 5 mm, a diameter of 1.5 mm, and ${{{{{{\rm{\lambda }}}}}}}_{0}$ = 2.65 µm is depicted in Fig. 2b. It shows a typical quadratic phase profile where the incremental rotation angle decreases towards the center of the metalens. A scanning electron microscopy (SEM) image of a final metallic polymer metalens is depicted in Fig. 2c, showing the excellent fabrication quality achievable with spin-coated PEDOT:PSS. As known from previous studies, the plasma frequency/wavelength, which defines the wavelength above which the polymer and thus the nanoantennas become metallic, is around ${{{{{{\rm{\lambda }}}}}}}_{{{{{{\rm{p}}}}}}}$ = 1.3 µm^11^. Consequently, the metallic polymer nanoantennas—the building blocks of the metalenses—exhibit a plasmonic resonance in the infrared (IR) spectral range, as depicted in Fig. 2d. The TM (transverse magnetic) resonance position of our used nanoantennas with a length of ${{{{{\rm{L}}}}}}$ = 380 nm, width of ${{{{{\rm{W}}}}}}$ = 160 nm, and height of ${{{{{\rm{H}}}}}}$ = 90 nm is at ${{{{{\rm{\lambda }}}}}}$ = 2.65 µm, used as working wavelength in all following experiments. Simulations of the plasmonic resonances for different nanoantenna lengths as well as field plots can be found in Fig. S2 in the Supplementary Information. In Fig. 2e we map the focus of a metallic polymer metalens along the optical axis when illuminated with RCP light (see Figure S3 in Supplementary Information for details on the exact measurement setup). The refractive power of the metalens manifests itself in a focus in the focal plane of the metalens, as depicted in Fig. 2e. Furthermore, the Gaussian beam profile in the focal plane is highly symmetric in both dimensions (${{{{{\rm{x}}}}}}$ and ${{{{{\rm{y}}}}}}$). This is proven by a cross-sectional profile along the ${{{{{\rm{x}}}}}}$-direction through the focus, as shown in Fig. 2f. We obtain a focal spot with a FWHM (full width at half max) of 81 µm at an illumination wavelength of ${{{{{\rm{\lambda }}}}}}$ = 2.65 µm. Please note that the theoretical diffraction-limited FWHM is on the order of 10 µm, which assumes perfect monochromatic illumination and neglects dispersion as well as aberrations. A potential reason for aberrations lies with the fabrication of our large-area polymer metalens, which is still comparably sensitive to fabrication inaccuracies. Such inaccuracies can, e.g., result from the write-field alignment of the electron-beam-lithography or from the semi-directional argon dry etching. Furthermore, the metalens with diameter 1.5 mm is designed from subunits of 100 µm × 100 µm write-fields. In consequence, this means that the polymer nanoantennas of the metalenses will show deviations from the ideal shape/size as well as from the exact position and rotation angle. These errors can introduce aberrations such as chromatic aberration, spherical aberration, coma, and astigmatism. Furthermore, we are at the resolution limit of our IR camera (pixel size 85 µm with 3× magnification; Effective measurement resolution in focal plane: 28.3 µm) which can cause further errors. So far, the focusing efficiency of our metallic polymer metalens is 0.8%. It is limited by the overall modulation of the plasmonic resonance, shown in Fig. 2d. Material engineering as well as optimizing the doping levels of the metallic polymers will enhance the focusing efficiency in the future.

**Fig. 3:**
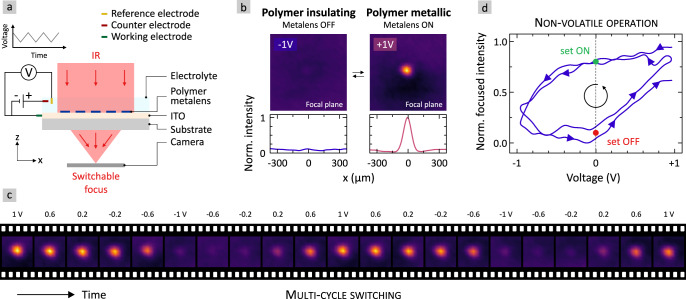
High-contrast electrical switching of a metallic polymer metalens-on-demand. **a** Setup to electrically switch a metalens. An electrochemical three-electrode setup is used to tune the applied voltage via cyclic voltammetry. The camera is placed in the focal plane of the metalens (focal length of ${{{{{\rm{f}}}}}}$ = 5 mm) to image the optical performance of the metalens. **b** Camera images and cross-sectional intensity profiles in the focal plane of the metalens. Left: An applied voltage of −1 V switches the polymer insulating and thus the metalens OFF with no diffractive power. Right: A voltage of +1 V switches the polymer metallic and the metalens ON. A focus is observed. **c** Time sequence of camera images showing the focused intensity by the polymer metalens while the applied voltage is switched via cyclic voltammetry between ±1 V (2 cycles). A video showing all frames and the cyclic voltammogram can be found in Movie S1 in the Supplementary Information. **d** Normalized focused intensity by the polymer metalens as a function of the applied voltage during the switching cycles shown in (**c**). The scanning direction is marked with arrows. Non-volatile operation is possible at 0 V. The polymer metalens remains in the ON state at 0 V (set ON, marked green) when previously +1 V was applied. In contrast, it remains OFF at 0 V (set OFF, marked red) when previously −1 V was applied.

Next, we turn our attention to the switching performance of the metallic polymer metalens. The electrical switching is carried out using an electrochemical setup in a liquid electrolyte, as it is sketched in Fig. 3a (see Methods for further information). It comprises a three-electrode setup including a reference and counter electrode^44^. Please note that the electrochemical cell can be completely air-sealed with UV-sensitive glue or thermoplastic^45^. To foster integration into AR/VR devices, the liquid electrolyte could also be replaced by a gel-like or solid electrolyte. In our configuration, the polymer metalens itself acts as the working electrode and is contacted via the ITO layer. The applied voltage is cycled between the metallic polymer state at +1 V and the insulating polymer state at −1 V via cyclic voltammetry. Simultaneously, the transmitted intensity and beam profile are imaged using the IR camera positioned in the focal plane of the metalens. Most importantly, we demonstrate in Fig. 3b that the polymer metalens can be switched between an ON- and OFF-state on demand depending on the applied voltage. A voltage of +1 V turns the plasmonic resonance of the nanoantennas and thus the metalens ON (right). The polymer switches into its metallic state and we observe a focal spot in the focal plane. In contrast, an applied voltage of −1 V turns the plasmonic resonance and the metalens OFF (left). The polymer switches into the insulating state and the refractive power of the metalens is turned OFF. Almost the entire incident laser power will be in the co-polarized part of the transmitted light and is filtered with the circular analyzer. Please note that the refractive index of PEDOT:PSS in the insulating/dielectric phase is likely too low to be efficiently used as Mie resonator. Finally, the electro-optical switching between ON- and OFF-state is reversible and repeatable over hundreds of switching cycles with switching frequencies up to 33 Hz, as previously demonstrated^11^.

Selected images of two cycles of electrochemical metalens switching via cyclic voltammetry (CV) are depicted in Fig. 3c (see Movie S1 in Supplementary Information for all frames including the cyclic voltammogram). CV means that the applied voltage is slowly ramped up and down between ±1 V. The images show the intensity distribution in the focal plane for different applied voltages during the switching cycles. The time axis is from left to right. The scan rate is 20 mV/s. The refractive power of the metalens is reversibly switched ON and OFF. The focused intensity is high at +1 V and low at −1 V. Interestingly, the metalens remains in the metallic ON-state for voltages down to −0.2 V. This is observable even better in the graph depicted in Fig. 3d. Here, we plot the normalized focused intensity over the applied voltage for the two full cycles. The scanning direction is indicated with arrows. In the backward scan from +1 V to −1 V, we find, that the focused intensity remains almost constant and thus the metalens remains in the ON-state down to voltages of −0.2 V. The intensity fades starting at applied voltages below −0.3 V and the metalens is switched OFF at −1 V. In contrast, in the forward scan from −1 V to +1 V the metalens remains in the OFF-state up to 0 V. From this point the focused intensity increases again until it reaches it maximum value around +1 V. The typical degradation of the optical modulation of our polymer metasurfaces is on the order of 25% after 290 switching cycles^11^. These values are measured via chronoamperometry (CA), where the applied voltage is quickly changed between +1 V and −1 V (square-wave voltage) with frequencies of 1 Hz and 30 Hz. Figure 3d shows that the normalized focused intensity by our metalens drops by 35% after only 2 cycles of cyclic voltammetry (CV). However, as mentioned, in such CV measurements the applied voltage is slowly ramped up and down between ±1 V. Thus, one cycle of CV in Fig. 3d takes almost 4 min compared to less than a second in CA. As the degradation scales with the total time of applied voltage, the degradation in Fig. 3d is already significant after 2 long cycles of CV. Potential sources for the degradation of the polymer nanoantennas are the volume expansion during the switching as well as irreversible reactions during electrochemical oxidation and reduction.

Due to the shown hysteresis in Fig. 3d, the metalens can either be set ON or set OFF at 0 V (marked in green and red) depending on the preceding applied voltage.  Thus, an energy-efficient non-volatile operation is possible. The dwell time in the insulating OFF state after the external voltage has been turned off is 2–3 min until the polymer returns and relaxes into its pristine metallic state. Consequently, the dwell time in the metallic ON state is infinity as it is identical to the pristine state. This principle is used in the following to switch and set the states of a multi-functional metaobjective comprising two electrically switchable metallic polymer metalenses-on-demand.

**Fig. 4:**
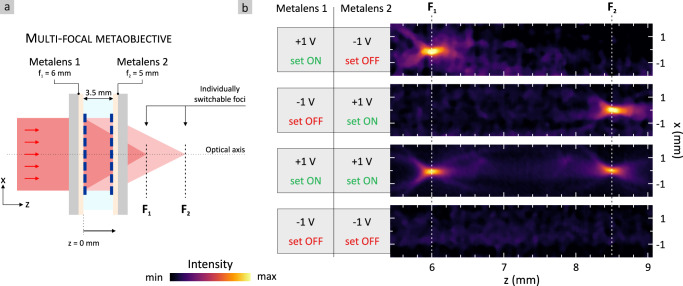
Non-volatile electrically switchable multi-functional metaobjective. **a** Schematic drawing of the arrangement of the metaobjective. It comprises two polymer metalenses spaced with an electrolyte. Both are electrically switchable separately via an applied voltage. The metaobjective is illuminated with an IR laser beam (${{{{{\rm{\lambda }}}}}}$ = 2.65 µm) and the focused intensity is imaged along the optical axis. **b** The metaobjective from metallic polymer allows for four different states. Non-volatile operation is used to set the refractive power of the individual metalenses either ON or OFF with an applied voltage of +1 V or −1 V, respectively. State 1 (metalens 1: ON, metalens 2: OFF): single focus at focal plane ${{{{{{\rm{F}}}}}}}_{1}$ of metalens 1. State 2 (metalens 1: OFF, metalens 2: ON): single focus at focal plane ${{{{{{\rm{F}}}}}}}_{2}$ of metalens 2. State 3 (metalens 1: ON, metalens 2: ON): two foci at focal planes ${{{{{{\rm{F}}}}}}}_{1}$ and ${{{{{{\rm{F}}}}}}}_{2}$. State 4 (metalens 1: OFF, metalens 2: OFF): no refractive power of metaobjective. The entire metaobjective is thus switched OFF.

The polymer metaobjective is sketched in Fig. 4a. As mentioned, it consists of two metalenses fabricated from metallic polymer, which are separately switchable. The focal lengths are ${{{{{{\rm{f}}}}}}}_{1}$ = 6 mm for metalens 1 and ${{{{{{\rm{f}}}}}}}_{2}$ = 5 mm for metalens 2 and they are spaced by 3.5 mm. Both metalenses comprise nanoantennas (${{{{{\rm{L}}}}}}$ = 380 nm, ${{{{{\rm{W}}}}}}$ = 160 nm, ${{{{{\rm{H}}}}}}$ = 90 nm) resonant at ${{{{{\rm{\lambda }}}}}}$ = 2.65 µm, which is used as the laser illumination wavelength. The metalenses are mounted in the same electrochemical cell and their gap is filled with electrolyte (see Methods and Fig. S4 in the Supplementary Information). We use a non-volatile operation (as explained in Fig. 3d) to set the individual states of the two metallic polymer metalenses.

The metaobjective is switchable between four different states. The results are depicted in Fig. 4b. We plot a cross-section of the ${{{{{\rm{xz}}}}}}$-plane which is obtained by moving the IR camera along the optical axis (${{{{{\rm{z}}}}}}$-direction). In the first state (top) metalens 1 is set ON (+1 V) whereas metalens 2 is set OFF (−1 V). Only metalens 1 focuses the incoming circularly polarized light beam and we find a single focus in the focal plane ${{{{{{\rm{F}}}}}}}_{1}$ at position z = 6 mm. The second state (metalens 1: set OFF, metalens 2: set ON) switches the focal length of the metaobjective and we observe a single focal spot in the focal plane ${{{{{{\rm{F}}}}}}}_{2}$ of metalens 2 at z = 8.5 mm. Furthermore, we realize two unusual states with our metallic polymer metaobjective. Switching both metalenses simultaneously ON via an applied voltage of +1 V creates a multi-focal metaobjective. We obtain focal spots in both focal planes ${{{{{{\rm{F}}}}}}}_{1}$ and ${{{{{{\rm{F}}}}}}}_{2}$. An applied voltage of −1 V to both polymer metalenses (bottom of Fig. 4b) sets both metalenses OFF and into the insulating state. The refractive power of the entire metaobjective is switched OFF and no focal spots are observed. It should be noted that in the ON/ON-state the focusing (conversion) efficiency of each metalens defines how the two metalenses in the metaobjective influence each other. For a low focusing efficiency, one obtains two foci (compare state 3 in Fig. 4b). In this case, the rear metalens will focus the non-converted circularly polarized light. Thus, the intensity of the rear focus depends on the converted intensity and thus focusing efficiency of the front metalens. Ultimately, for a conversion/focusing efficiency of 100%, the two foci in the ON/ON state will vanish. Rather the rear metalens will convert and further focus (or collimate) the light coming from the front metalens. In this case, the metalenses would act similar to conventional lenses.

In conclusion, we have demonstrated electrically switchable high-contrast metalenses-on-demand and a multi-functional metaobjective by using the optical metal-to-insulator transition in metallic polymer nanoantennas. We observe an ON- and OFF-state of the metalenses, where their refractive power can be switched ON and OFF via an applied voltage of +1 V and −1 V, respectively. These low switching voltages are CMOS compatible and switching can be operated up to video rate frequencies of 33 Hz^11^. Further improvements of the switching speed might be achievable, for example, by either changing the overall size of the nanoantennas (less material to be switched), by removing the SiO_2_ cover on top of the nanoantennas (larger surface for electrolyte to access), or by using different electrolytes (e.g., smaller ions compared to TBAPF_6_). By combining two metallic polymer metalenses into a metaobjective, four different states including a multi-focal state were demonstrated. In the future, several directions are possible which will allow to further push the integration of metallic polymer metasurfaces into electro-optical devices. For example, development in the material properties and fabrication processes will optimize and enhance the switching speeds, lifetime^46^, and efficiency to meet the requirements of future display technologies. Mass producibility of the metalenses is possible, e.g., via nanoimprint-lithography (roll-to-roll) to meet the requirements in consumer-based products. In AR/VR devices, our metalenses could specifically be used in the NIR or MIR for, e.g., eye-tracking devices, facial recognition, or to scan and create a 3D map of the surrounding, similar to future miniaturized LiDAR devices in autonomous cars. Furthermore, we aim at even higher charge carrier densities and thus shorter plasma wavelengths to enable switchable plasmonic operation in the telecom or even visible range. With these developments, even visible video-rate holograms for AR and VR applications should come in reach.

## Methods

### Spectral IR measurements

We use a Fourier-transform infrared (FTIR) spectro-microscopy setup (Bruker Vertex 80 spectrometer with Hyperion 3000 microscope) to measure the spectral response of the metallic polymer nanoantennas and metalenses. ITO-covered glass substrates are used as reference.

### Electrochemical switching of metalenses/metaobjective

A home-built electrochemical (EC) cell with optical access is used to electrically switch the metallic polymer metalenses/metaobjective. The cell is sealed on the top and bottom with the samples (polymer metalenses on ITO-covered glass substrate) to facilitate measurements in transmission (see Figure S4 in the Supplementary Information). The EC cell is filled with an electrolyte (0.1 mol/l TBAPF_6_ in acetonitrile). We use a three-electrode setup where the voltage is controlled via a potentiostat (BioLogic SP-200). The ITO layer (for electrical contacting) and the polymer metalenses/metaobjective serve as the working electrode. The counter electrode (platinum wire) and the reference electrode (silver/silver-chloride wire) are in direct contact with the electrolyte. The electrical wiring for the metaobjective comprising two polymer metalenses is explained and sketched in Fig. S4 in the Supplementary Information.

Please note that our concept is based on an electrochemical approach where the conducting electrolyte is uniquely chosen to oxidize and reduce our metallic polymer PEDOT:PSS at ±1 V. ITO will not show any redox reaction for this particular voltage range. Similarly, there will be no charge accumulation at the top surface of ITO, as the adjoining layers are electrically conducting, and a current is flowing through the electrochemical cell. A change of the optical properties of ITO via gating and charge accumulation occurs only in a MOS (metal-oxide-semiconductor) configuration, which means that the ITO (serving as ground) and the counter electrode (e.g., gold or aluminum layer) are separated by an insulating layer^9^. Consequently, we do not expect the ITO to change its optical properties in our specific sample geometry.

## Supplementary information

Supplementary Information

Description of Additional Supplementary Files

Supplementary Movie 1

## Data Availability

The authors declare that all data supporting this work are contained in graphics displayed in the main text or in the Supplementary Information. Data used to generate these graphics are available from the authors upon request.
