# Supplementary material for: Electro-active metaobjective from metalenses-on-demand
Source: Nat Commun. 2022 Nov 23;13:7183. doi: 10.1038/s41467-022-34494-0 (PMC9684136; doi:10.1038/s41467-022-34494-0)
Supplement: Supplementary file 1 — Supplementary Information [file 41467_2022_34494_MOESM1_ESM.pdf]

**Supplementary Information**

**for**

# Electro-active metaobjective from metalenses-on-demand

*Julian Karst[1], Yohan Lee[1], Moritz Floess[1], Monika Ubl[1], Sabine Ludwigs[2], Mario Hentschel[1], and Harald Giessen[1]*

[1] 4th Physics Institute and Research Center SCoPE, University of Stuttgart, Pfaffenwaldring 57, 70569 Stuttgart, Germany

[2] IPOC-Functional Polymers, Institute of Polymer Chemistry, & Center for Integrated Quantum Science & Technology (IQST), University of Stuttgart, Pfaffenwaldring 55, 70569 Stuttgart, Germany

| | Approach | Material Examples | Stimulus | Required Voltage | Process | Switching Resonance ON-OFF (Metal-to-Insulator Transition) | Operation in | Conversion Efficiency Metasurface | Switching speed / frequency | High pixel density possible (<1 µm) + individual address. | Exemplary Publications |
|---|---|---|---|---|---|---|---|---|---|---|---|
| **Direct approaches** (nanoantenna material is switched) | Electro-chemical metallic polymer (this work) | PEDOT:PSS | Voltage | ±1 V | Metal-to-insulator transition (electrochemical) | Yes Contrast 100% | Transm. | 1% | 33 Hz | Yes | This work, [1] |
| | Hydrogen-sensitive metals | Magnesium | Gas (hydrogen) | - | Metal-to-insulator transition | Yes Contrast 100% | Transm. & Reflect. | 23% (comb. with Au) | Minutes | No | [2–7] |
| | (Electro-)thermal materials | GST | Voltage (resistive heating) | 5-25 V | Refractive index tuning (structural phase transition) | No (resonance tuning) | Reflect. | 8% | 10 kHz range | Not known (depends on heat dissipation) | [8,9] |
| **Indirect approaches** (surrounding material is switched) | Electro-chemical polymers | PANI, PEDOT, ProDOT | Voltage | ±1 V (depends on polymer) | Refractive index tuning (electrochemical and Pockels-effect) | No (resonance tuning) | Transm. & Reflect. | Depends on resonant metasurface | 1-50 Hz | Yes | PANI: [10–12]<br>PEDOT: [13,14]<br>ProDOT: [15] |
| | Electro-mechanical MEMS | Silicon | Voltage | 2-80 V | Mechanical movement | No | Transm. & Reflect. | Depends on resonant metasurface | 10-100 kHz range | No | [16,17] |
| | Electro-mechanical elastomers | Polyacrylate | Voltage | 3 kV | Mechanical movement | No | Transm. & Reflect. | Depends on resonant metasurface | 30 Hz | No | [18] |
| | Electro-active liquid crystals | Liquid crystals | Voltage | 1-50 V | Refractive index tuning (crystal reorientation) | No (resonance tuning) | Transm. & Reflect. | Depends on resonant metasurface | 100 Hz | Yes | [19–22] |
| | Electro-active ITO | Indium-Tin-Oxide | Voltage | ±5 V | Refractive index tuning (charge accumulation) | No (resonance tuning) | Reflect. | Depends on resonant metasurface | 1-5 MHz | Yes | [23,24] |
| | (Electro-)thermal materials | GST, $VO_2$, PDMS | Voltage, Temperature | 5-12 V | Refractive index tuning (structural phase transition) | No (resonance tuning) | Transm. & Reflect. | Depends on resonant metasurface | GST: 10 kHz, $VO_2$: 1-5 Hz PDMS: 5 Hz | Not known (depends on heat dissipation) | GST: [25,26]<br>$VO_2$: [27]<br>PDMS: [28] |

**Table S1. Comparison of different approaches for switchable and active metasurfaces.**

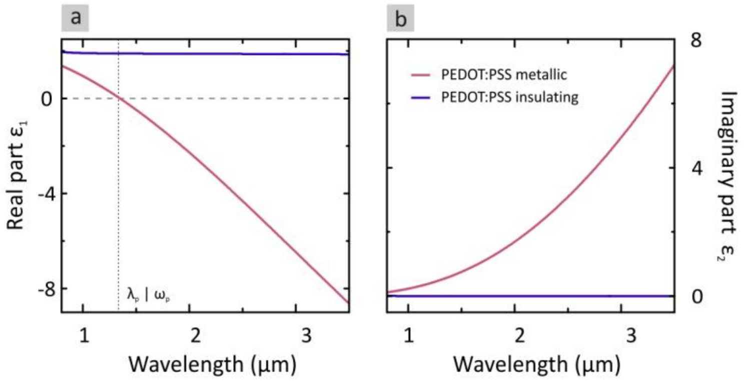


**Figure S1. Dielectric function of PEDOT:PSS (J. Karst et al. Science 374, 612 (2021)).** (a) Real part $\varepsilon_1$ of PEDOT:PSS in the metallic state (red) and insulating state (blue). The crossing where $\varepsilon = 0$ in the metallic state is marked and defines the plasma frequency $\omega_p$ / plasma wavelength $\lambda_p$ above which PEDOT:PSS has metallic properties. (b) Imaginary part $\varepsilon_2$ of PEDOT:PSS again in the metallic (red) and insulating state (blue).

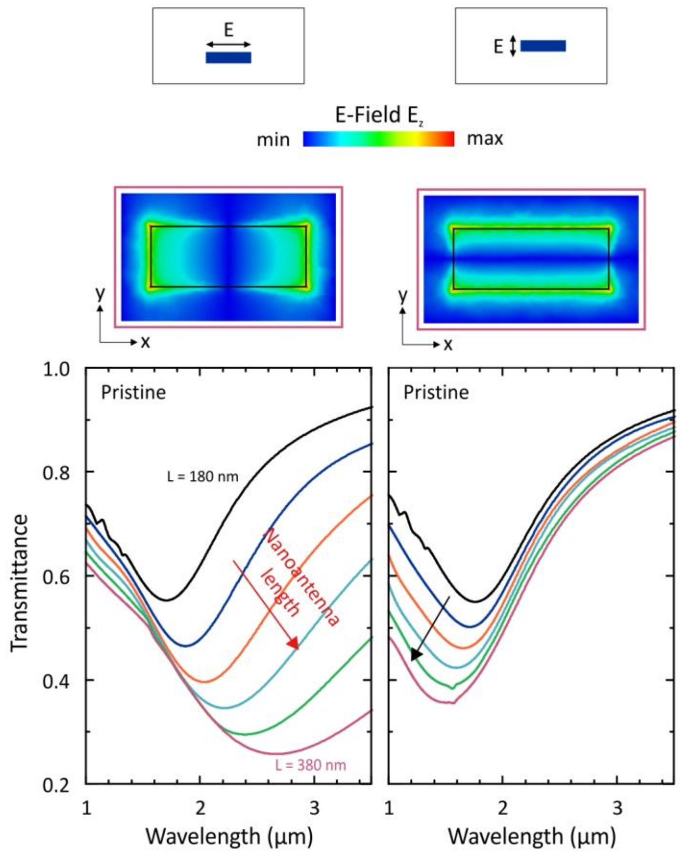


**Figure S2. Simulated plasmonic resonances of metallic polymer nanoantennas.** Bottom: Simulated transmittance as function of wavelength for different nanoantenna lengths for TM (left) and TE polarized light (right). The length is tuned from L = 180 nm to L = 380 nm, the width is W = 160 nm, the height is H = 90 nm. Top: Field plots of the z-component of the electric field for the nanoantennas with length L = 380 nm. The plots show the near-field distribution for TM (left) and TE (right). The simulations are obtained via the frequency solver of CST Microwave Studio.

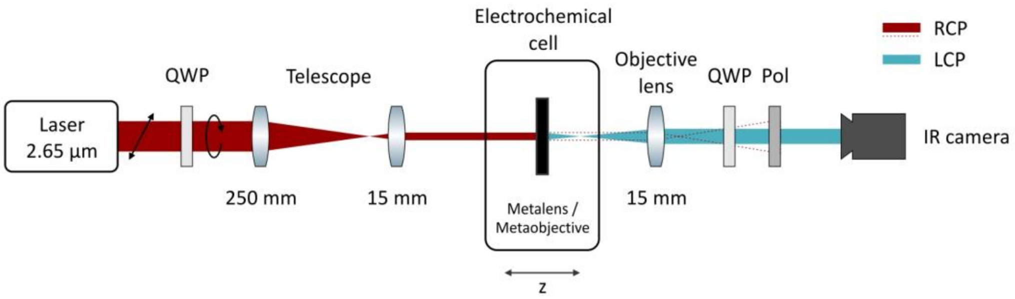


**Figure S3. Setup to measure and map the focus of the metalenses and the metaobjective in Figures 2-4 of the main manuscript.** We use a laser (Stuttgart Instruments Alpha HP) tuned to 2.65 µm to be resonant with the plasmonic metallic polymer nanoantennas. The laser output is linearly polarized. Using a quarter-waveplate (QWP) we obtain right-circularly polarized (RCP, red) light. A telescope reduces the beam diameter to illuminate only the metalens/metaobjective (diameter 1.5 mm). The metalens/metaobjective is mounted inside an electrochemical cell to facilitate electrical switching. The transmitted (focused) light is polarization converted and is left-circularly polarized (LCP, blue). The transmitted light is collected and imaged to an IR camera (Spiricon Pyrocam III) using an objective lens. The remaining fundamental RCP light is filtered using a circular analyzer (combination of QWP and polarizer). By moving the entire electrochemical cell along the optical axis in z-direction, we can map the focus of the metalens/metaobjective. Please note that the metalenses show identical functionality when being illuminated with LCP light instead of RCP light. Furthermore, the coherence length of our pulsed laser is on the order of 300-500 µm. As the distance of our two metalenses (3.5 mm) in the metaobjective is much longer, we expect that there is no influence of any interference effect on the scattered signals from each metalens. Furthermore, we do not observe interference effects in our optical measurements as seen in Figure 4b.

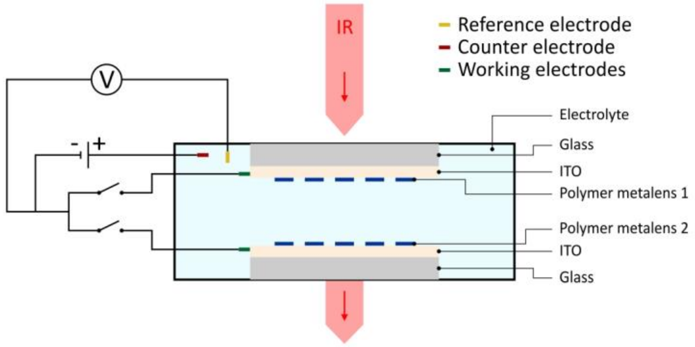


**Figure S4. Schematic setup to switch the individual metalenses of the metaobjective in Figure 4 of the main manuscript.** Both polymer metalenses are mounted inside an electrochemical cell filled with electrolyte. We use ITO covered glass substrates to allow electrical addressability and operation in transmittance. Both ITO layers are contacted separately. Using switches, we can choose to which metalens the set voltage is applied to. Furthermore, we can also apply the same voltage to both polymer metalenses simultaneously to set both metalenses to the either ON- or OFF-state.

## Supplementary References:


1. Karst, J. *et al.* Electrically switchable metallic polymer nanoantennas. *Science* **374**, 612–616 (2021).
2. Sterl, F. *et al.* Magnesium as novel material for active plasmonics in the visible wavelength range. *Nano Lett.* **15**, 7949–7955 (2015).
3. Strohfeldt, N. *et al.* Yttrium hydride nanoantennas for active plasmonics. *Nano Lett.* **14**, 1140–1147 (2014).
4. Duan, X., Kamin, S. & Liu, N. Dynamic plasmonic colour display. *Nat. Commun.* **8**, 14606 (2017).
5. Duan, X. & Liu, N. Scanning plasmonic color display. *ACS Nano* **12**, 8817–8823 (2018).
6. Yu, P. *et al.* Generation of Switchable Singular Beams with Dynamic Metasurfaces. *ACS Nano* **13**, 7100–7106 (2019).
7. Yu, P. *et al.* Dynamic Janus Metasurfaces in the Visible Spectral Region. *Nano Lett.* **18**, 4584–4589 (2018).
8. Zhang, Y. *et al.* Electrically reconfigurable non-volatile metasurface using low-loss optical phase-change material. *Nat. Nanotechnol.* **16**, 661–666 (2021).
9. Wang, Y. *et al.* Electrical tuning of phase-change antennas and metasurfaces. *Nat. Nanotechnol.* **16**, 667–672 (2021).
10. Kaissner, R. *et al.* Electrochemically controlled metasurfaces with high-contrast switching at visible frequencies. *Sci. Adv.* **7**, eabd9450 (2021).
11. Peng, J. *et al.* Scalable electrochromic nanopixels using plasmonics. *Sci. Adv.* **5**, eaaw2205 (2019).
12. Xu, T. *et al.* High-contrast and fast electrochromic switching enabled by plasmonics. *Nat. Commun.* **7**, 1–6 (2016).
13. Ratzsch, J. *et al.* Electrically switchable metasurface for beam steering using PEDOT polymers. *J. Opt.* **22**, 124001 (2020).
14. Zhang, S., Feng, L., Zhang, H., Liu, M. & Xu, T. Electrochromic modulation of plasmonic resonance in a PEDOT-coated nanodisk metasurface. *Opt. Mater. Express* **10**, 1053 (2020).
15. Xiong, K. *et al.* Video Speed Switching of Plasmonic Structural Colors with High Contrast and Superior Lifetime. *Adv. Mater.* **33**, 2103217 (2021).
16. Arbabi, E. *et al.* MEMS-tunable dielectric metasurface lens. *Nat. Commun.* **9**, (2018).
17. Holsteen, A. L., Cihan, A. F. & Brongersma, M. L. Temporal color mixing and dynamic beam shaping with silicon metasurfaces. *Science* **365**, 257–260 (2019).
18. She, A., Zhang, S., Shian, S., Clarke, D. R. & Capasso, F. Adaptive metalenses with simultaneous electrical control of focal length, astigmatism, and shift. *Sci. Adv.* **4**, 1–8 (2018).
19. Badloe, T., Kim, I., Kim, Y., Kim, J. & Rho, J. Electrically Tunable Bifocal Metalens with Diffraction-Limited Focusing and Imaging at Visible Wavelengths. *Adv. Sci.* **8**, 2102646 (2021).
20. Li, S. Q. *et al.* Phase-only transmissive spatial light modulator based on tunable dielectric metasurface. *Science* **364**, 1087–1090 (2019).

21. Li, J., Yu, P., Zhang, S. & Liu, N. Electrically-controlled digital metasurface device for light projection displays. *Nat. Commun.* **11**, 3574 (2020).

22. Franklin, D., Frank, R., Wu, S. T. & Chanda, D. Actively addressed single pixel full-colour plasmonic display. *Nat. Commun.* **8**, 1–10 (2017).

23. Shirmanesh, G. K., Sokhoyan, R., Wu, P. C. & Atwater, H. A. Electro-optically Tunable Multifunctional Metasurfaces. *ACS Nano* **14**, 6912–6920 (2020).

24. Park, J. *et al.* All-solid-state spatial light modulator with independent phase and amplitude control for three-dimensional LiDAR applications. *Nat. Nanotechnol.* **16**, 69–76 (2021).

25. Yin, X. *et al.* Beam switching and bifocal zoom lensing using active plasmonic metasurfaces. *Light Sci. Appl.* **6**, e17016 (2017).

26. Abdollahramezani, S. *et al.* Electrically driven reprogrammable phase-change metasurface reaching 80% efficiency. *Nat. Commun.* **13**, 1696 (2022).

27. Kim, Y. *et al.* Phase Modulation with Electrically Tunable Vanadium Dioxide Phase-Change Metasurfaces. *Nano Lett.* **19**, 3961–3968 (2019).

28. Afridi, A. *et al.* Electrically Driven Varifocal Silicon Metalens. *ACS Photonics* **5**, 4497–4503 (2018).
