# Supplementary material for: Electro-active metaobjective from metalenses-on-demand
Source: Nat Commun. 2022 Nov 23;13:7183. doi: 10.1038/s41467-022-34494-0 (PMC9684136; doi:10.1038/s41467-022-34494-0)
Supplement: Supplementary file 2 — Description of Additional Supplementary Files [file 41467_2022_34494_MOESM2_ESM.pdf]

Description of Additional Supplementary Files

Supplementary Movie 1: Video of continuous electrochemical switching of metalens. Top: Camera images of focal plane of a metallic polymer metalens recorded during electrochemical switching (cyclic voltammetry). It shows the refractive power to turn ON and OFF depending on the applied voltage. Bottom: Cyclic voltammogram (current against applied voltage) of the electrochemical switching (cyclic voltammetry).
